# Spatial perspective and identity in visual awareness of the bodily self-other distinction

**DOI:** 10.1038/s41598-023-42107-z

**Published:** 2023-09-11

**Authors:** Tommaso Ciorli, Lorenzo Pia

**Affiliations:** 1 SAMBA (SpAtial, Motor and Bodily Awareness) Research Group, Department of Psychology, University of Turin, Via Verdi 10, 10123 Turin, Italy; 2 NIT (Neuroscience Institute of Turin), Turin, Italy

**Keywords:** Consciousness, Perception, Human behaviour

## Abstract

Spatial perspective and identity of visual bodily stimuli are two key cues for the self-other distinction. However, how they emerge into visual awareness is largely unknown. Here, self- or other-hands presented in first- or third-person perspective were compared in a breaking-Continuous Flash Suppression paradigm (Experiment 1) measuring the time the stimuli need to access visual awareness, and in a Binocular Rivalry paradigm (Experiment 2), measuring predominance in perceptual awareness. Results showed that, irrespectively of identity, first-person perspective speeded up the access, whereas the third-person one increased the dominance. We suggest that the effect of first-person perspective represents an unconscious prioritization of an egocentric body coding important for visuomotor control. On the other hand, the effect of third-person perspective indicates a conscious advantage of an allocentric body representation fundamental for detecting the presence of another intentional agent. Summarizing, the emergence of self-other distinction into visual awareness would strongly depend on the interplay between spatial perspectives, with an inverse prioritization before and after conscious perception. On the other hand, identity features might rely on post-perceptual processes.

## Introduction

The ability to distinguish between the own body and the body of another person is a key signature of human nature, and necessary for survival. Indeed, self-other distinction of corporeal stimuli is a prerequisite for a wide range of cognitive functions such as motor control, social perception, and interactions with the others^1,2^. Such skill is known to rely on a complex interplay between a variety of sensorimotor signals, so that their optimal integration allows to successfully attribute a given bodily stimulus to the self or to another person. It is worth noticing that among all the sensory domains, there is a modality that seems to have a more prominent role, and it is vision^3^. For this reason, understanding how the visual system differentially processes self- and other- bodily stimuli represent one of the most significant scientific challenges for human sciences.

Extracting relevant visual information from human bodies allows us to build a variety of important judgements as, for instance, those related to age, ethnicity, gender, and so on. Among these, there is also the ability to attribute the seen body/body parts as belonging to oneself or, alternatively, to another person. Within an experimental context, one way to scientifically investigate the self-other distinction for corporeal stimuli in visual modality is manipulating the spatial perspective in which the stimulus appears. The rationale behind this idea is that a human body part, the hand for instance, can potentially be perceived from a first-person perspective (i.e., upright orientation with fingers up), or in a third-person perspective (i.e., upside down orientation, with fingers downward). Within this framework, it has been demonstrated that hand images depicted in the first-person perspective are naturally attributed to one’s own body, whereas those in the third-person perspective are referred to the body of someone else^4–6^. Interestingly, a number of neuroimaging studies reported different patterns of cortical activation that underlies the visual processing of hand stimuli coded in the two perspectives^7–9^. This, in turn, suggests the possible functional dissociation between the two perspectives. A second option to examine the self-other distinction for visually presented hands is, much more obviously, to vary the identity of the stimulus that, according to its peculiar visual features, is attributed to oneself or to another person. Within this other line of research, it has been shown that encoding of the own hand and that of another person is underpinned by substantially different patterns of brain activity^10–13^, and they can be selectively disrupted by means of experimentally controlled virtual lesions^14,15^. Within a neuropsychological approach, it has been reported that brain lesions to the right hemisphere can disrupt the so-called self-advantage (i.e., the facilitation to implicitly discriminate self-versus other people's visually presented hands^16,17^). Moreover, brain damages can lead also to the misattribution of the own contralesional limb to another person^18,19^, or even the opposite behavior, namely the misattribution of somebody else’s arms to oneself^20–22^. As above, also these data suggest that the stimulus identity (i.e., self or other) might be functionally dissociable.

The abovementioned considerations identify spatial perspective and identity as the two crucial cues to successfully assigning a seen hand to the own body or to the body of somebody else. However, how these two features differentially affect the information flow from early levels of unconscious visual processing to a complex conscious representation of the self-other distinction is largely unknown. Given innate social attitude of the human species, understanding whether these cues can influence self-other distinction even unconsciously, or rather, whether they necessarily require visual awareness is a fundamental scientific challenge. Moreover, to the best of our knowledge, there is only one published study that addressed the relation between visual awareness and self-other distinction^23^. That work reported that multisensory integration subserving the attribution of the own body parts to the self actually promoted visual awareness. To fill this gap, here we focused on the role of perspective and identity in the prioritization of hand stimuli in visual awareness. In particular, we examined two distinct aspects of visual awareness, namely the access to and the dominance in. As regards the access to visual awareness, we employed the breaking-Continuous Flash Suppression (bCFS) paradigm firstly reported by Jiang and coworkers^24^. Here, a high-contrast dynamic mask flashed to one eye effectively suppresses a target stimulus of increasing intensity presented to the other eye. By decreasing the contrast of the mask over the trial, the suppressed stimulus becomes visible and the time required to detect it reflects the moment in which it gains access to awareness^25^. With respect to the dominance in visual awareness, we employed the Binocular Rivalry (BR) paradigm where the two stimuli presented each to one eye simply compete for visual awareness which alternates the conscious representation of the two rival stimuli back and forth during the trial. The momentarily perceived and reported stimulus at the conscious level is called dominant, the other is said to be suppressed^26^, being momentarily unreported and perceived without awareness. Higher stimulus predominance over the other stimulus (i.e., being consciously perceived for longer time) reflects conscious visual awareness prioritization, rather than initial unconscious prioritization of bCFS. In summary, in the present study we compared self-hand stimuli vs. other-hand stimuli presented in first-person perspective vs. third-person perspective, and we measured both the time of access (Experiment 1) and the time of dominance (Experiment 2) in visual awareness. The datasets analyzed during the current study are available from the corresponding author (L.P.) on reasonable request. In line with the literature suggesting possible functional dissociations between perspective and identity, we predicted that they could be differentially prioritized in visual awareness.

## Experiment 1

### Materials and methods

We employed the bCFS paradigm to test whether and how spatial perspective (first-person or third-person), and identity (self or other) affected the time of the access to visual awareness of corporeal stimuli (hands).

#### Participants

22 (15 F, mean age = 24.5 ± 2.5 years) participants with normal or corrected-to-normal vision were administered the experiment after having provided a written informed consent. Since previous work showed that participants are advantaged in the processing of the dominant self-hand^6^, we recruited both right-handed (19) and left-handed (3) participants (self-report). They were all naïve to the research questions underneath the study, which was approved by the Ethical Committee of the University of Turin (protocol n. 0486683) and performed in accordance with relevant guidelines of the declaration of Helsinki. Initial sample size (n = 27) was based on similar studies using such paradigms^27^, and we later estimated the statistical power of our results in a post-hoc power analysis with g*Power (3.1.9.7).

#### Apparatus, stimuli, and procedure

At the arrival, each participant’s dominant hand was photographed with a digital camera in a controlled setting. The picture was taken from above in the first-person perspective. Then it was black-and-white transformed, cropped with Photoshop (2019), and a 180°-rotated copy was created. A same-laterality and same-gender hand from the previous participant was used as ‘other’ stimulus and shown in advance for visual familiarity acquisition. Thus, target stimuli were self/other hands in first/third-person perspective (2 × 2 design).

**Figure 1:**
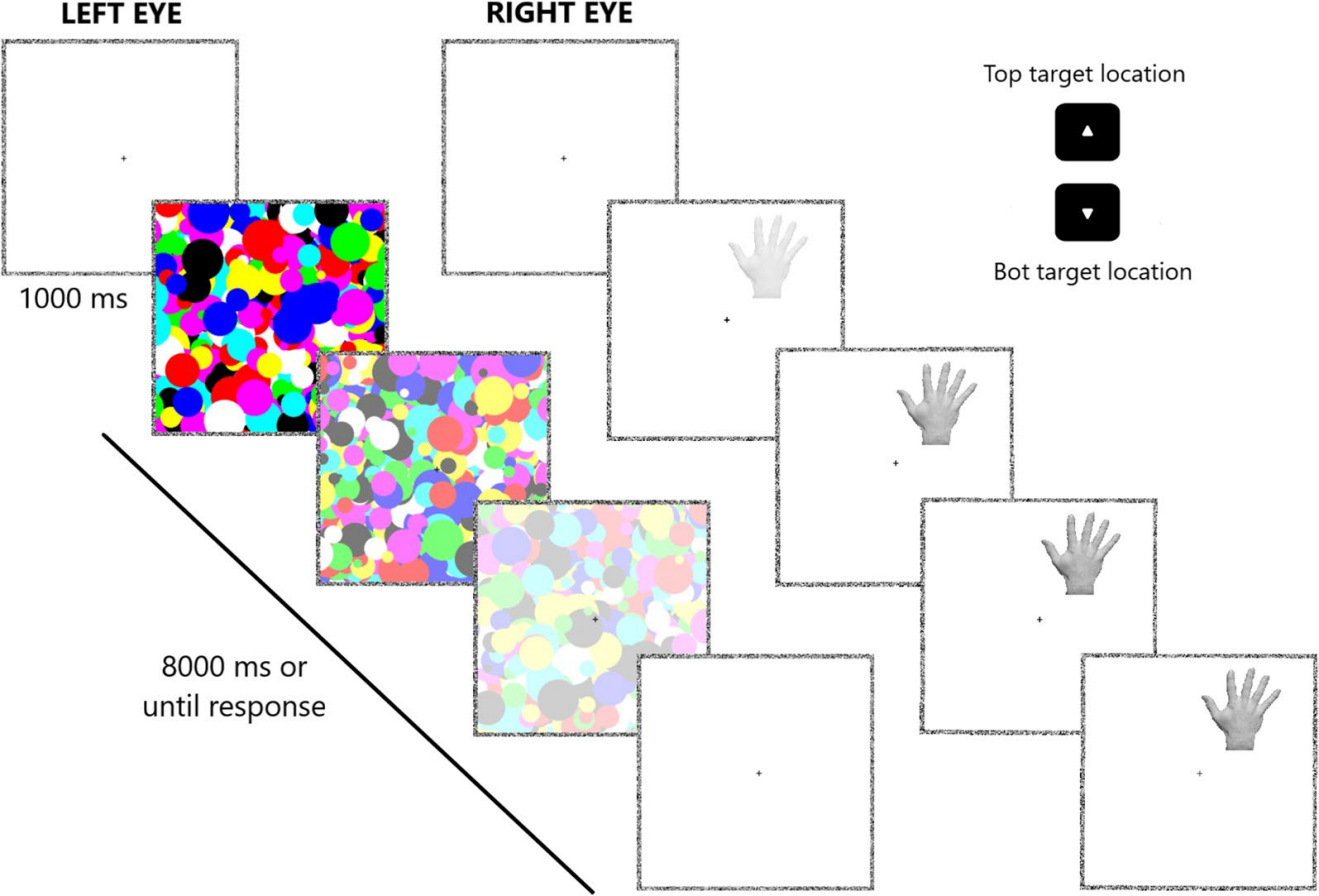
Schematic representation of the bCFS trial. After 1 s of inter-trial-interval, a high-contrast mask updating at a frequency of 10 Hz is shown to one eye, and its transparency was increased from 0 to 100% within 1–7 s after the end of the ITI. The target hand is shown to the other eye, and its transparency is decreased from 100 to 0% within the first second of trial. Each trial lasted for a maximum of 8 s or until response (top-arrow, top location; bottom-arrow, bot location) once participants detected even a part of the target breaking the suppression, pressing the corresponding arrow as fast as possible.

The experiment was programmed with MATLAB (Release 2021b) and Psychtoolbox^28^, and 3° × 4.2° target stimuli were presented on a BenQ Monitor (1.920 × 1.080 pixel resolution, 120 Hz, 24″) at a distance of approximately 57 cm. Participant’s head was stabilized by a chinrest with a custom built-in stereoscope, allowing for stable binocular vision once adjusted for each participant. The target stimulus was presented to one eye and a dynamic high-contrast Mondrian-pattern mask (i.e., randomly arranged circles of distinct colors, and sizes between 0.3° and 1.2°) was flashed at 10 Hz to the other. Both the stimulus and the mask were contained in a fusion square (11.7° × 11.7°, one per eye, each at a distance of 5.8° from the center) made of noise-pixels (width 0.2°). The screen background was black, while the fusion squares’ area was white with a black fixation cross in the center. For each trial, the target stimulus was shown by linearly decreasing its transparency from 100 to 0% within the first second of trial, presenting it at the top or the bottom of the fusion square (with a random horizontal jitter). At the same time, the transparency of the high-contrast mask presented to the other eye was linearly increased from 0 to 100% within 7 s (starting after the first second of trial). Instructions were presented in a written and verbal form, and 8 practice trials were employed to familiarize with the task. Participants were asked to respond as fast and accurately as possible by localizing the position of the target through the keyboard arrows (i.e., top position: up arrow key, bottom position: down arrow key), once visible. They were also required to answer even if they simply had a strong feeling that something more than the mask was present (i.e., preventing conscious perception of stimuli). During each trial (lasting until response or for a maximum of 8 s), they were asked to keep the eyes on the central fixation cross, to avoid active gaze-searching, to prevent both blinking and closing one eye at a time (which were allowed in the 1 s ITI). Within each of the four conditions, stimuli were randomly administered 24 times to the right eye in a top position, 24 times to the right eye in a bottom position, 24 times to the left eye in a top position, and 24 times to the left eye in a bottom position for a total of 96 trials. The four conditions were administered to each participant in a randomized order for a total of 384 trials. After 128 and 256 trials, a small break was made (see Fig. 1 for time course of a trial and stimuli).

### Statistical analysis

Participants reporting unstable binocular perception (N = 0), those with accuracy lower than 90% (N = 4), and those with a mean value ± 2.5 SD from the group mean (N = 1) were excluded, so that the final sample consisted of 22 participants. Trials with response time lower than 300 ms (0.35% of the trials) were excluded since they indicated that stimuli were not suppressed. Then, for each of the four conditions, mean response times for corrected responses only were calculated, and then log-transformed to account for a not normal distribution of the data (Shapiro Wilks < 0.05). We reported both frequentist statistics and Bayes factors (BFs; Cauchy distribution = 0.707) by using JASP (JASP Team, 2016). We conducted a 2 × 2 repeated-measures ANOVA with the factors Identity (self/other) and Perspective (first/third). For significant effects, we assessed to what extent the evidence supported the alternative hypothesis model (BF_10_). For non-significant results, we assessed to what extent the evidence supported the null hypothesis model (BF_01_).

### Results

**Figure 2:**
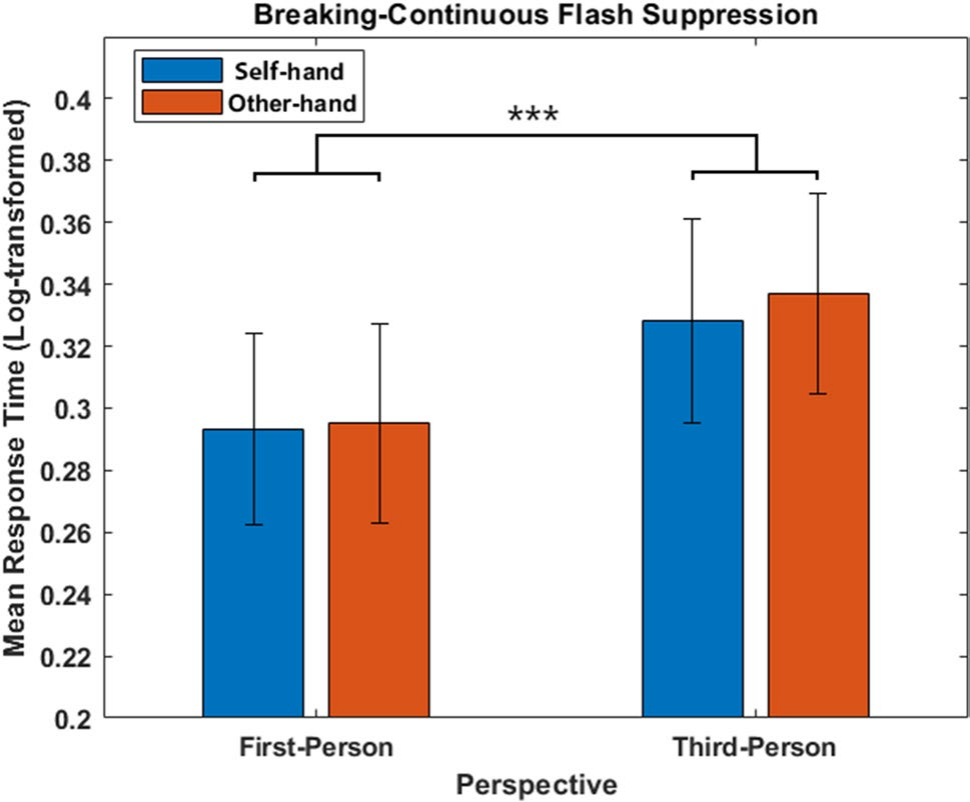
Results of Experiment 1. Mean log-transformed response time as a function of hand perspective and identity (and SE), reflecting the suppression time of the targets. *p < 0.05, **p < 0.01, ***p < *0.001.*

Mean response times for correct response before the log-transformation were 2.49 s (SE =  ± 0.17) in the *self first-person* condition, 2.71 s (SE =  ± 0.18) in the *self third-person* condition, 2.50 s (SE =  ± 0.18) in the *other first-person* condition, and 2.75 s (SE =  ± 0.19) in the *other third-person* condition. The repeated-measures ANOVA on log-transformed means for correct responses revealed a main effect of perspective (F_(1,21)_ = 17.49, *p* < 0.001, η_p_^2^ = 0.454; BF_10_ = 74.27), with significantly (*t* = -4.182, *p* < 0.001, Cohen’s d = − 0.89; BF_10_ = 76.61, very strong evidence in favor of the alternative hypothesis) faster responses to *first* (mean = 0.29, SE =  ± 0.031), than *third* (mean = 0.33, SE =  ± 0.032) *person* perspectives. The main factor identity (*p* = 0.53; BF_01_ > 100, very strong evidence for the null hypothesis) and the interaction perspective x identity (*p* = 0.59; BF_01_ = 8.70) were not significant. See Fig. 2 for the results. We then performed a post-hoc power analysis, from which we observed the 99% of statistical power to detect the effect of perspective in a two-tail t-test with our sample size, alpha (0.05), and effect-size (*dz* = 1.2) determined by their mean values and standard deviations.

Summarizing, these results show that first-person, with respect to the third-person spatial perspective, speeded up the access to visual awareness for hands stimuli.

## Experiment 2

### Materials and methods

We employed the BR paradigm to evaluate whether spatial perspective (first-person or third-person), and identity (self or other) of corporeal stimuli (hands) influenced the dominance in visual awareness of corporeal stimuli (hands).

#### Participants

24 (15 F, mean age = 25.08 ± 3.36 years) right or left-handed participants (self-report) with normal or corrected-to-normal vision were administered the experiment after having signed the written informed consent. They were all naïve to the research questions underneath the study, which was approved by the Ethical Committee of the University of Turin (protocol n. 0486683) and performed in accordance with relevant guidelines of the declaration of Helsinki. Sample size was similarly based on studies using such paradigms^27^, and we post-hoc estimated the observed statistical power of our results with g*Power (3.1.9.7).

#### Apparatus, stimuli, and procedure

The laboratory setting, the apparatus, and the stimuli construction were the same as in Experiment 1.

**Figure 3:**
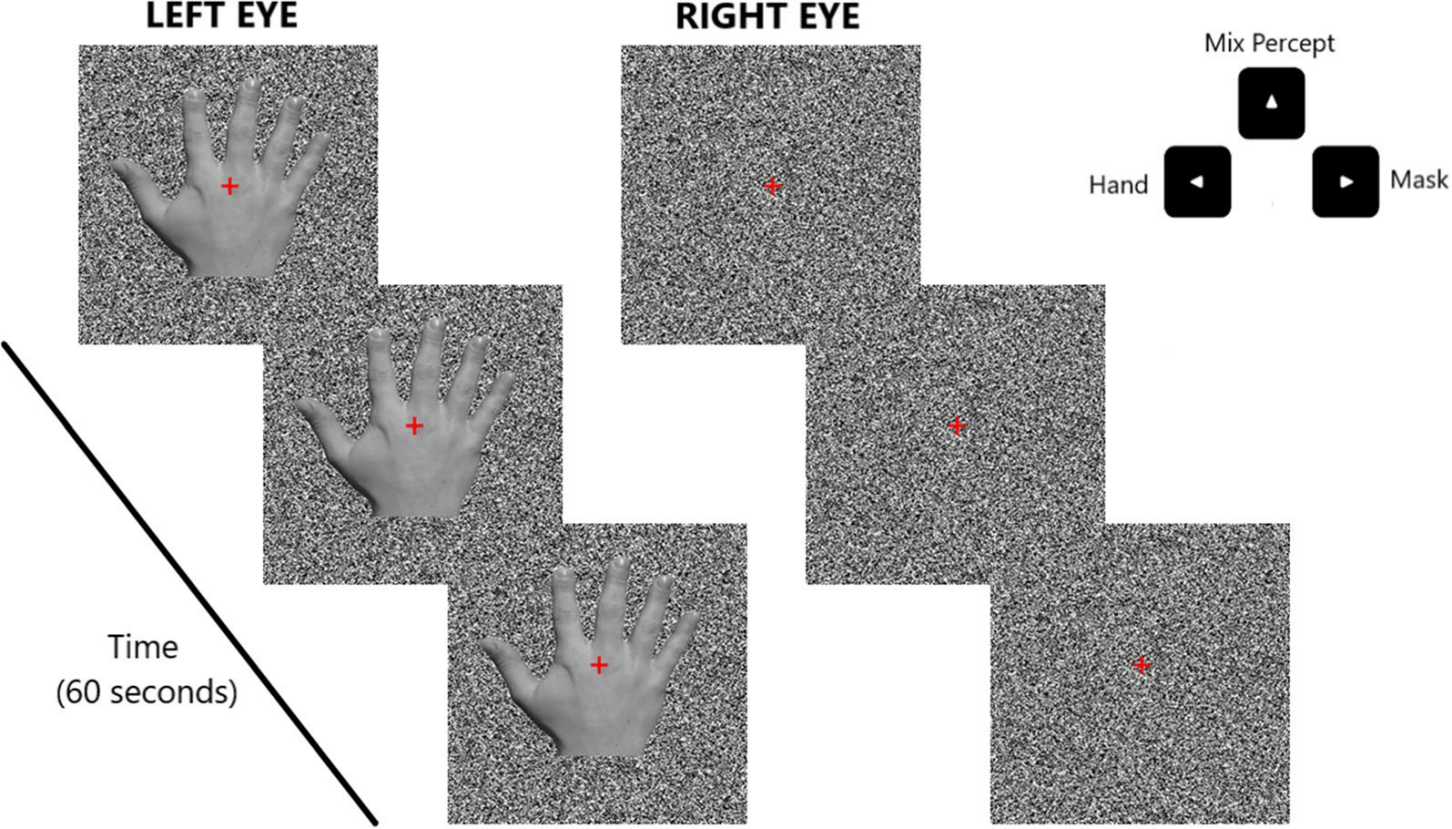
Schematic representation of the Binocular Rivalry trial. Participants were exposed for 60 s to binocular presentation of the target hand in one eye, and a static noise-pixel mask to the other. They had to report their dominant percept and the changes over time, by pressing the correspondent arrow (left-arrow, hand predominance; right-arrow, mask predominance; top-arrow, not a predominance). After each trial there was 30 s of eye-rest.

A static noise-pixel mask was presented to one eye, whereas the target stimuli were shown to the other eye, for 60 s. Targets were superimposed onto the same noise background in order to avoid blank spaces and dominance incongruencies. Stimuli were shown in two identical squared contours (10° × 10°) consisting of black and white pixels (width 0.2°) at a distance of 5° from the center (one on the left, one on the right) and with a red central fixation-cross in the middle. Outside the contours, the background was black. In a random half of the trials, targets were shown to the left eye, in the other half to the right. Instructions were presented in a written and verbal form, and with 4 practice trials participants were familiarized with the task. They were asked to look at the fixation cross during the trials to avoid eye-blinking and closing one eye. They had to focus on the target, and to press the left arrow if they perceived more than the 50% of the hand (i.e., target predominance), the right arrow if they saw less than the 50% (i.e., mask predominance), and the up arrow if they did not perceive a predominance (i.e., mixed percepts). Importantly, for all the trial duration, they were asked to promptly report their percept each time it changed (in the direction of the change). Within each of the four conditions, stimuli were randomly administered 12 times to the right eye, and 12 times to the left eye for a total of 24 trials. Between each trial there was a 30 s break for eye resting, whereas after 12 trials there was a longer (minimum 60 s) break (see Fig. 3).

### Statistical analysis

Participants with extremely short dominance for targets and/or reported instable binocular perception (N = 2), and those with mean values out of the mean ± 2.5 SD (N = 1) were excluded so that the final sample consisted of 24 participants. Mean response times within each condition, for both the stimuli and for the mask, were calculated (data were normally distributed according to a Shapiro–Wilk test, *p* > 0.05). As a common procedure in BR studies, mixed dominance times were not considered for the analysis. Then, we extracted the predominance ratio per each condition, namely the difference between the cumulative duration of the target and the corresponding cumulative duration of the mask, divided by the sum of the two: $x_{ratio} = \frac{{\left( {x_{target} - x_{mask} } \right)}}{{\left( {x_{target} + x_{mask} } \right)}}$. On such dependent variable, we employed JASP (JASP Team, 2016) to run a 2 × 2 repeated-measures ANOVA with the factors Identity (self/other) and Perspective (first/third).

### Results

**Figure 4:**
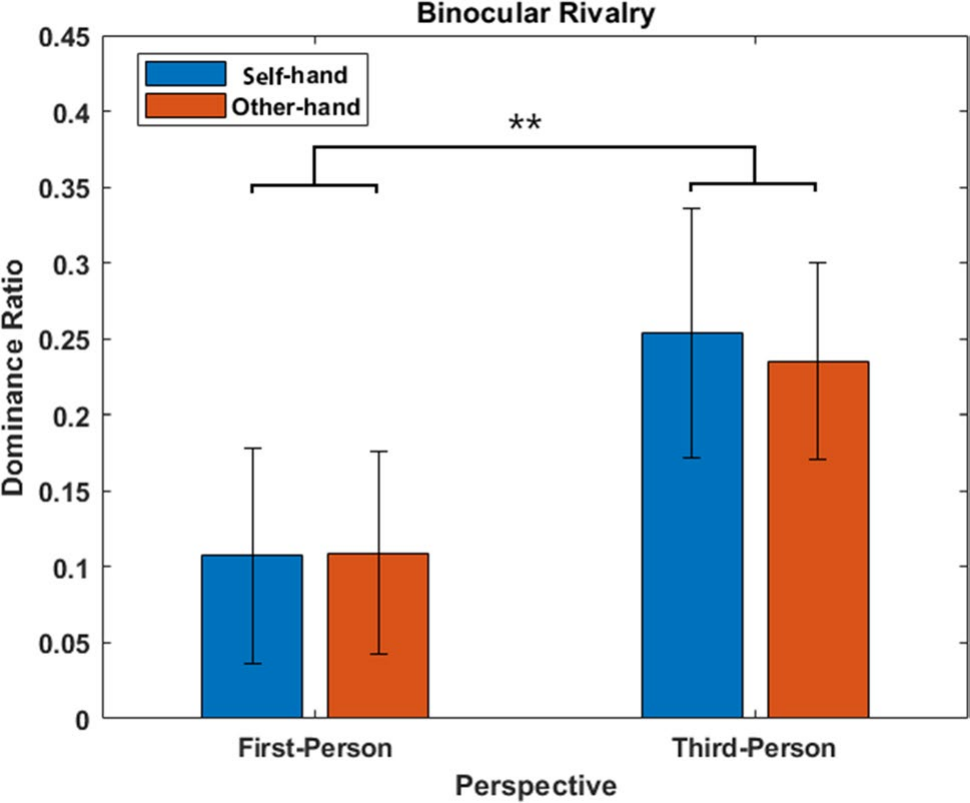
Results of Experiment 2. Mean cumulative dominance ratio (and SE) for each condition, scored as the cumulative dominance of the target minus the cumulative duration of the mask, divided by the sum of the two measures. *p < 0.05, **p < 0.01, ***p < *0.001.*

Mean response time for the targets was 27.47 s (SE =  ± 1.87 s), while the mask mean was 19.57 s (SE =  ± 1.96 s), showing that hand stimuli were perceived for more time than the pixel mask. Among the targets, the repeated-measures ANOVA revealed a significant main effect of *perspective* (F_(1,23)_ = 5.567, *p* < 0.05, η_p_^2^ = 0.195; BF_10_ = 2.42) with the t-test indicating that participants reported greater predominance (*t* = − 2.359, *p* < 0.05, Cohen’s d > 0.40; BF_10_ = 2.12) for *third* (mean = 28.79 s, SE =  ± 2.11) than first-person (mean = 26.15 s, SE =  ± 1.78) perspective. No other significant differences emerged from the analysis; the factor identity relied on *p* = 0.57 with a BF_01_ = 8.14, whereas the interaction among identity x perspective *p* = 0.48 and BF_01_ = 9.5. We repeated the analysis on the dominance ratio per each condition, revealing, again, a main effect of *perspective* (F_(1,23)_ = 9.957, *p* < 0.01, η_p_^2^ = 0.302; BF_10_ = 11.15, strong evidence) showing significant greater responses for *third-* than first-person perspectives (*t* = − 2.359, *p* < 0.05, Cohen’s d > 0.40; BF_10_ = 9.67). Again, the factor identity was not significant (*p* = 0.79, BF_01_ = 38.77) nor the interaction (*p* = 0.71, BF_01_ = 12.02). See Fig. 4 for the results. From the post-hoc power analysis we observed that we had the 53% of statistical power to detect the effect of perspective in a two-tail t-test considering our sample size, alpha (0.05), effect-size (*dz* = 0.41) and their mean values with standard deviations.

Summarizing, these results show that third-person, with respect to first-person spatial perspective, dominated for more time the visual awareness for hands stimuli.

## Discussion

In the present study, we investigated the role of spatial perspective and identity in the emergence into visual awareness of the self-other distinction for bodily stimuli (i.e., hands). We capitalized on stimulus prioritization by focusing on the time of access (breaking-Continuous Flash Suppression, Experiment 1), and on the time of dominance (Binocular Rivalry, Experiment 2). In both experiments, we compared self-hand stimuli vs. other-hand stimuli presented in the first-person perspective vs. the third-person perspective. Our results show that only spatial perspective promoted the stimuli within visual awareness. Specifically, the first-person perspective speeded up the access, while the third-person perspective increased the perceptual dominance.

With respect to conscious access timing (Experiment 1), it is important to elucidate the nature and the rationale of the employed paradigm (i.e., bCFS). Whenever a high salient image is projected to one eye, and a low salient image to the other, the latter is suppressed from conscious perception. Within the experimental context, the salient stimulus is usually a high-contrast Mondrian-like visual pattern (i.e., geometrical shapes usually varying every 100 ms in terms of color and spatial arrangement), whereas the non-salient stimulus (i.e., the target) is often a static, low-contrast image. This paradigm is termed Continuous Flash Suppression (CFS)^29^ and it has been created to study unconscious visual processing. One of its variants, the so-called breaking Continuous Flash Suppression (bCFS^25^), measures the time the target needs to break the suppression induced by the mask. The key assumption is that stimulus categories that overcome interocular suppression (also known as breakthrough times) faster enabling access to visual awareness receive a prioritized early perceptual processing before awareness^30^. In other words, bCFS employs a direct measure of subjective conscious perception to infer differential unconscious processing, namely the time a given stimulus needs to be detected. It is important to note, however, that this paradigm has some criticalities. It is known that subjective and objective measures of visibility, for instance, can be anatomo-functionally dissociated^31^, which may produce incongruent results. Moreover, any subjective RT-based method cannot precisely disentangle whether the response is purely due to perceptual detection or if also identification plays a role^32^. Additionally, disparities in the low-level visual features and/or response bias can alter breakthrough times, thereby compromising the validity of the technique for studying unconscious processes. Capitalizing on such evidence, the scientific community also relied on non-speeded accuracy-based methods with objective awareness measures^30,33,34^. However, this does not imply that the bCFS should be discarded or does not convey theoretical values. In Experiment 1, we showed that the visual image of a hand presented in the first-person perspective broke the suppression faster than the same visual image, with identical low-level features, presented in the third-person, regardless of its identity. In other words, hand’s perspective was differentially processed before consciousness, with the first-person perspective being prioritized. How can we explain this result? Firstly, the effect of visual perspective per se is not trivial but, rather, consistent with a number of studies with the same paradigm suggesting that not only low-level, but also high-level stimulus properties can be processed before consciousness (i.e., something typically thought not to be possible without awareness; see^35–37^ for discussions on this topic). For instance, it has been demonstrated that face orientation (e.g.,^24,38,39^), facial emotional content (e.g.,^40–42^), eye gaze direction^43,44^, familiarity and emotional valence of words^45^, multimodal congruency^46^, and degree of natural content of the visual scene^47^ can modulate the suppression time. Some of these high-level effects have been replicated also with more objective and accuracy-based techniques (e.g., face orientation^34^ and eye gaze direction^48^). However, others were not, being confounded by differences in low-level features^49,50^, decision criteria^33^, and object-scene integration^51,52^, highlighting how such factors might impact on conscious access measures misinterpreting the scopes and the limits of unconscious perception. But what about the interpretation of the specific access to awareness advantage of the first-, with respect to the third-, person perspective that we reported here? One of the already mentioned neuroimaging studies (i.e., Chan et al.^8^) manipulated both visual perspective and identity of human body images. Among the various pattern of brain activations, the work reported that a specific brain structure (i.e., the left Superior Parietal Lobule SPL) was significantly more activated in first-, than in third-, person perspective. Interestingly, other studies investigating the neural correlates of representing the environment from egocentric perspectives in a broader sense reported the same selective activation^53–55^. Such set of evidence seems to be in line with the known role of the SPL in representing nearby stimuli in body-centered coordinates^56^. Importantly, these representations in SPL are thought to be largely unconscious^57^ because of their roles that mostly deal with visuomotor control of actions. In other words, such unconscious nature guarantees a quick integration of visual corporeal inputs over eye fixations. This, in turn, would allow the generation of stable visuospatial representation of the own body regardless of the ongoing saccades and attentional focus. According to all these considerations, here we put forward the idea that our findings might represent the behavioral counterpart of such unconscious processing of egocentric body representations. Such a direct body coding, without a continuous update of the eye position, could be further optimized if irrelevant information is suppressed. Hence, the absence of any identity effect that we report here could simply reflect an optimization procedure. Interestingly, in their paper, Chan and colleagues^8^ examined also the role of identity of bodily stimuli (i.e., self vs other) and did not find any differential activation at the level of the SPL.

As regards the conscious perceptual dominance investigated in Experiment 2, even here we must clarify better the nature of the BR paradigm. It is known that the brain typically does not unify different images seen by the two eyes in a single visual percept. Within the experimental setting, this phenomenon is evident in the BR paradigm in which two images are presented to the two eyes. Conscious perception alternates back and forth the representation of the two stimuli, including periods of mixed percepts. Such alternation represents the competition between the images for conscious representation, with the perceived and reported stimulus being called dominant, and the other unperceived and unreported one suppressed^26^, and such competition is the product of multiple and continuous interactions between lower-level and higher-level brain areas^58^. BR has been widely used to study both low-level and higher-level effects on visual awareness^26,59^, with the idea that stimuli being reported for longer time (i.e., perceptually dominant and more likely to be consciously perceived compared to the rival stimulus) are prioritized in conscious visual awareness. Experiment 2 showed that the visual image of a hand presented in a third-, with respect to first-, person perspective dominated visual perception for longer time. As discussed for Experiment 1, the effect of perspective per se adds to other evidence on the role of higher-order stimulus features in the dominance time, such as stimulus context (e.g.,^60^), multimodal congruency (e.g.,^46^), cognitive salience (e.g.,^61^), and voluntary attention (e.g.,^62^). The specific effect of the third-person perspective we found here, it suggests that hand’s spatial perspective differentially affects conscious visual awareness that prioritize bodily stimuli that most likely reflect other agents, underlying a stronger tuning with social cognition compared to self-centered stimuli that were found in Experiment 1. This process would increase the salience of bodily stimuli in a perspective in which the existence of another human beings ready to act is likely. Then, this information would be necessarily integrated with more complex processes dealing with social cognition that attract our attentional and conscious resources. The absence of any identity effects might indicate that those features would be accounted for later at higher cognitive levels. Interestingly, two neuroimaging studies^7,8^ reported that the Extrastriate Body Area (EBA), a brain region involved in body perception of non-facial body parts, resulted to be significantly associated with stimuli presented in the third-, rather than in the first-, person perspective. These data have been interpreted as a result of the fact that this area is preferentially attuned to the perception of others’ bodies, and more specifically its function relies on detecting the presence of an intentional actor through the visual analysis of the form of other human bodies^63^. Nonetheless, the EBA is part of a more complex brain network subserving higher-level social cognition^64^. According to all these considerations, we propose that our findings would represent the behavioral counterpart of such conscious perceptual enhancement of allocentric body representations. Interestingly, some of the previously mentioned studies examined the EBA sensitivity to the identity features (i.e., self vs. other) of bodily stimuli, without reporting any significant effects^8,11,12^; but see also^13^. Hence, the null effect of identity on perceptual dominance is in line with this evidence and supports the idea that the EBA might be blind to factors such as ownership/identity.

Summarizing, here we demonstrated that spatial perspective, but not identity, differentially affected visual awareness prioritization, with an unconscious advantage for the first-, and a conscious advantage for the third-person perspective. Capitalizing on the existing literature, we argue that unconscious prioritization of the first-person perspective would represent at the behavioral level a mechanism that supports action monitoring through the generation of a stable visuospatial body representation. Moreover, this process would be optimized by suppressing irrelevant information such as, for instance, stimulus identity. On the other hand, the conscious prioritization of the third-person perspective would be the behavioral counterpart of a mechanism useful to detect potential intentional actors in the environment. Only later this information would be integrated with other cues (e.g., identity) to support social cognition. Taken together, these considerations indicate that the self-other distinction for visually presented bodily stimuli seems to be strongly rooted in the interplay between spatial perspectives displaying an inverse prioritization before and after conscious perception. Those cues can be sufficient in a large variety of daily life situations, so that identity would be involved only when strictly necessary, perhaps at more post-perceptual and higher cognitive levels. Interestingly, such ‘suppression’ of identity features is also adaptive, in a way that perceptual hand features might change (i.e., while wearing gloves), but the perspective in which you perceive yourself or another person remain stable. Interestingly, this is in line with the literature on bodily illusions based on multisensory integration. These experimental manipulations show that external objects as, for instance, rubber hands^65–68^ or virtual bodies^69–71^, can be misattributed to oneself. Crucially, this illusory experience occurs if bodily stimuli are presented in the first-, but not in the third-person perspective, and this happens regardless of its visual appearance (i.e., identity).

Before concluding, we must acknowledge some limitations, as well as future directions for novel studies. A first possible problem regards the above-mentioned criticalities reported for the bCFS paradigm. As regards the possible role of low-level features we believe this is unlikely since, apart from the fact that the stimuli creation was performed under rigorous experimental rules, low-level features were identical for the two spatial perspectives, being the same stimulus rotated by 180 degrees, and self-hand stimuli were used as other-hand stimuli for the subsequent participant (i.e., we alternated the same stimuli, keeping the same low-level features and only changing their high-level meaning in the eye of the observers). As for the other problems (i.e., subjective/objective measures of visibility, detection/identification processes and response bias), we cannot affirm they did not affect our results. Hence, more stringent techniques (e.g., dissociation paradigms^34^) could be employed to overcome these limitations. A second possible limitation is related to the so-called inversion effect reported for faces and bodies^72^. This effect consists in the reduced ability to visually recognize an object shown upside-down since that object entails an intrinsic canonical orientation. However, hands do not seem to have such a default representation (i.e., we are visually familiar with upside-down hands representing others), and, if that was the case, one would expect the same upright advantage in both bCFS and BR, rather than opposite patterns. As regards the possible future directions, we believe that forthcoming studies should try to clarify at which stage and to which extent visual features subserving identity processing occur. Indeed, whereas our findings suggest that such property does not affect visual awareness prioritization, others reported a self-advantage in an implicit self-other discrimination of hands stimuli^17^, and others more showed that accuracy and speed were higher for the own, with respect to other, face^73^. Given that we did not provide any neural evidence in support of our results, direct neural correlates of the hypothesized differential processing of the SPL and the EBA in unconscious and conscious perception are barely required. Interestingly, very recent findings pinpoint that, at least anatomo-functionally, the SPL and the EBA reciprocally exchange information useful to support the interface between body perception and motor processes^74^.

## Data Availability

The datasets analyzed during the current study available from the corresponding author (L.P.) on reasonable request.
